# Remarks on the Influence of the Brunonian System on the Practice of Medicine

**Published:** 1799-03

**Authors:** 


### *Remarks on the Influence of the Brunonian System on the Practice of Medicine, by Professor* HUFELAND.

IN the fourth volume of "*The Journal, exhibiting the Practice of Medicine and Surgery,*" publiſhed at Jena, we obſerve a curious eſſay, deſigned as an anſwer to the following ſingular and intereſting queſtion:—"What influence has the ſyſtem of JOHN BROWN hitherto had on the practice of medicine; and in what particular reſpects has it changed, improved, or vitiated the medical art?" The ingenious author of this eſſay, Profeſſor HUFELAND, (who is likewiſe the Editor of the Journal abovementioned) admits, in concurrence with many other eminent phyſicians in Germany and England, that the inventor of this doctrine was a man of conſiderable genius, and that his theory is replete with novel and excellent ideas; notwithſtanding which, it by no means merits the name of a *ſyſtem,* as it every where preſents evident chaſms and defects. The *conſtituent* part of medicine, as an art, muſt neceſſarily reſt on the obſervation of facts, or what we call experience; theory is of ſervice

ſervice merely in the *regulative* part of it, and muſt invariably accommodate itſelf to freſh modifications and changes, whenever experience ſhall pronounce them neceſſary. The Brunonian doctrine appears very plauſible and conſiſtent in theory, but is liable to this material objection, that it frequently and eſſentially diſagrees with matters of fact and experience. The principal point, therefore, to be conſidered is, whether the Brunonian mode of repreſenting ſubjects in medicine, has a tendency to facilitate the acquiſition of medical knowledge, and to improve the method of curing diſeaſes?—The learned Profeſſor ſeems inclined to put a negative on this queſtion; and obſerves, that Brown's diviſion of diſeaſes into *ſthenic* and *aſthenic*, is only *apparently* ſimple and eaſy, but that it is in *reality* a matter of conſiderable doubt and difficulty to diſtinguiſh them from one another with preciſion; and that there are certain diſtempers, in which it is almoſt impoſſible to trace and diſcover the ſymptoms of the ſthenic and aſthenic conſtitution. It is further difficult to eſtabliſh clearly, where there is *direct*, and where there is *indirect* debility; to aſcertain to what degree this ſubſiſts in the body, and determine what ſpecies of ſtimulus ought to be applied to it. In our opinion, medicine can derive little *poſitive* advantage from the multiplication of theories, however ingeniouſly framed, if they be not founded on the baſis of actual obſervation and experience. Inſtead of indulging the modern rage for *generalization*, we ought previouſly to collect a ſufficient number of analogous facts; and, being in poſſeſſion of theſe, we might gradually and cautiouſly venture to reduce them to particular claſſes, orders, &c. But as this reſult preſuppoſes long and attentive inveſtigation, by a cool, perſevering, and unprejudiced mind, (circumſtances and qualifications but rarely united in *one* individual) there is little hope of ſeeing a *theory* of medicine, or a *Syſtem of Noſology* eſtabliſhed, which in the preſent progreſſive ſtate of medical and phyſical ſcience, will be found of ſuch unperiſhable materials, as to ſtand the teſt of ages,

### *Additional Remarks on the preceding Subject, by a Jena Reviewer.*

Brown was unqueſtionably a man of an enterpriſing temper, and peculiar boldneſs: a man of genius; one who thought freely for himſelf, diſregarding mere authorities and opinions, however ſanctioned by the venerable *imprimatur* of time. Had he been ſo fortunate, by a favourable concurrence of circumſtances, as to eſtabliſh the ſtability of his principles on extenſive obſervation and clinical experience, he would indeed have rendered eſſential ſervices to medicine, and attained the praiſe of uncommon merit in this ſcience But whoever is anxious to obtain celebrity, and to contribute *real*

aſſiſtance aſſiſtance to the *practice of the healing art*, will find it no eaſy matter to accompliſh this deſirable end by ſolitary diſquiſitions in his ſtudy-room: he muſt range through a circle of patients; examine and conſult with them; and theſe again, in like manner, with him. Old and experienced practitioners will readily diſcover whether the author or founder of a ſyſtem be in fact a ſtranger to the diſeaſes he attempts to define or arrange; if in only a few inſtances they eſpy his weak ſide, and find his account of the progreſs of a diſeaſe inconſiſtent with the path of nature, his pretenſions are inſtantly decried, and his whole ſyſtem is placed on the condemned liſt. Brown was a *luxuriant genius*, and his medical eccentricities frequently exhibit ſomewhat of a marvellous, if not even a monſtrous appearance. We may, however, eaſily underſtand how it happens that this *ſoi-diſant* ſyſtem is now ſo fondly careſſed and honoured with approbation, eſpecially by young practitioners, before they can have treaſured up a fund of original experience; as, thus fortified, they approach the bed of the patient with a certain conſcious air of veteran firmneſs. In the aphoriſtical doctrines of Bruno, they find every ſubject of this complicated art treated in a much eaſier, more conciſe, and convenient manner, than in the old-ſtanding authorities of former ages; inſtead of ſtudying, in well-arranged elementary treatiſes, the nature of every diſeaſe, according to its different ſtages, ſymptoms, &c. and making themſelves acquainted with methods of cure adapted to the particular ſtate of the diſorder, as well as the peculiar conſtitution, temperament, and external conditions of the patient; they congratulate themſelves that ſuch diffuſeneſs is *now* perfectly unneceſſary, innumerable diſeaſes being claſſed under one head, and treated in a ſimilar manner, in this comprehenſive mode of claſſification; for inſtance, in hæmoptyſis, as well as in diarrhœa, hyſterics, &c. &c. Brown indiſcriminately recommends the uſe of chalybeates, rum, opium, and the like. This, ſurely will be more readily underſtood in theory, and followed in practice, than the old elaborate, or ſay pedantic diffuſeneſs, by which the ſtudy of medicine is rendered difficult to the tyro, and the practice of it puzzling, if not baffling to the beginner.

